# Cyclophosphamide and epirubicin induce high apoptosis in microglia cells while epirubicin provokes DNA damage and microglial activation at sub-lethal concentrations

**DOI:** 10.17179/excli2021-4160

**Published:** 2022-01-10

**Authors:** Rafael de la Hoz-Camacho, Ana Luisa Rivera-Lazarín, Jose Manuel Vázquez-Guillen, Diana Caballero-Hernández, Edgar Mendoza-Gamboa, Ana Carolina Martínez-Torres, Cristina Rodríguez-Padilla

**Affiliations:** 1 Universidad Autónoma de Nuevo León, Facultad de Ciencias Biológicas, Laboratorio de Inmunología y Virología, Monterrey 66455, Mexico; 2 LONGEVEDEN S.A. de C.V.

**Keywords:** microglia, breast cancer, neurotoxicity, chemotherapy, chemobrain, apoptosis

## Abstract

Chemotherapy Related Cognitive Impairment (CRCI), also called chemobrain, diminishes cancer patient's life quality. Breast cancer (BC) patients have been described to be importantly affected, however, the mechanism leading to CRCI has not been fully elucidated. Recent research proposes microglia as the main architect of CRCI, thus dysregulations in these cells could trigger CRCI. The aim of this research was to evaluate the effects of two drugs commonly used against breast cancer, cyclophosphamide (CTX) and epirubicin (EPI), on the microglia cell line SIM-A9, using the BC cell line, 4T1, as a control. Our results show that CTX and EPI decrease microglia-cell viability and increase cell death on a concentration-dependent manner, being 5 and 2 times more cytotoxic to microglia cell line than to breast cancer 4T1cells, respectively. Both chemotherapies induce cell cycle arrest and a significant increase in p53, p16 and γ-H2AX in breast cancer and microglia cells. Furthermore, mitochondrial membrane potential (ΔΨm) diminishes as cell death increases, and both chemotherapies induce reactive oxygen species (ROS) production on SIM-A9 and 4T1. Moreover, caspase activation increases with treatments and its pharmacological blockade inhibits CTX and EPI induced-cell death. Finally, low concentrations of CTX and EPI induce γ-H2AX, and EPI induces cytokine release, NO production and Iba-1 overexpression. These findings indicate that microglia cells are more sensitive to CTX and EPI than BC cells and undergo DNA damage and cell cycle arrest at very low concentrations, moreover EPI induces microglia activation and a pro-inflammatory profile.

## Introduction

Breast cancer (BC) is one of the main causes of death among women worldwide (American Cancer Society, 2017[1]). Treatments include surgery, radiotherapy, and chemotherapy. There are different types of drugs used as chemotherapy, of which two commonly used are anthracyclines and alkylating agents, such as epirubicin (EPI) and cyclophosphamide (CTX) (Feng et al., 2016[9]; Wu et al., 2016[46]). Most chemotherapies induce regulated cell death (RCD), a mechanism by which the cell activates its own machinery to self-destruct (Galluzzi et al., 2018[11]). Both chemotherapies, EPI and CTX, have similar action mechanisms that include DNA damage (Taymaz-Nikerel et al., 2018[40]), cell cycle arrest (Xiong et al., 2016[48]), mitochondrial alterations and oxidative stress (Prasad et al., 2010[31]), in different tumoral cell lines and some non-neoplastic cell lines (Standish et al., 2008[39]). Even though chemotherapies are effective against cancer cells, they also induce secondary effects, such as nausea, alopecia (Uchida et al., 2018[42]), hepatotoxicity, and cardiotoxicity (Wu et al., 2016[46]), directly diminishing patient's quality life. Immune cells are frequently damaged by cancer treatments (Verma et al., 2016[44]), either around the body or in specific organs, such as liver, lungs and recently associated, brain (Matsos et al., 2017[23]). 

In this regard, studies show that around 23 % of surviving chemotherapy-treated BC patients present cognitive impairment (de Ruiter et al., 2012[6]; Selamat et al., 2014[36]; Shen et al., 2019[37]; Ongnok et al., 2020[29]). Chemotherapy related cognitive impairment (CRCI) is known to appear after chemotherapy treatment of non-central nervous system (non-CNC) tumors, and recent studies propose microglia as the main architect of CRCI (Gibson et al., 2019[14]). Microglia are tissue resident macrophages that play vital functions on the central nervous system (CNS), such as homeostatic maintenance, release of neurotrophic factors and protection against pathogens. Because of their macrophage nature, microglia cells produce cytokines and regulate neuroinflammation, which has been associated with different neuropathologies, such as Parkinson's disease, Alzheimer's disease, and cognitive impairment (McLeary et al., 2019[25]). Studies have shown a decrease in white matter density in patients after chemotherapy, leading to further investigation on cell death in central nervous system cells (Matsos et al., 2017[23]). There is a gap in knowledge regarding the susceptibility of central nervous system cells to chemotherapies directed at non-CNS tumors. Although the central nervous system is protected by the blood brain barrier (BBB), increasing cytokines and ROS levels associated with cancer-induced inflammation have been proposed to augment its permeability (Yarlagadda et al., 2009[49]). Increased permeability of the BBB may allow the direct interaction between circulating drugs and central nervous system cells during cancer therapy. Thus, the aim of this study was to assess the sensibility of microglia cells to the antitumor drugs epirubicin and cyclophosphamide to provide evidence supporting role for chemotherapy in CRCI.

## Methods

### Reagents

Cells were maintained in DMEM-F12 and RPMI-1640 supplemented with 10 % fetal bovine serum (FBS) and 1 % penicillin-amphotericin-streptomycin (GIBCO by Life Technologies, Grand Island, NY) referred as complete DMEM-F12 or RPMI. FACS buffer is composed of 2 % of fetal bovine serum (FBS) in phosphate buffer saline 1X (PBS) at pH 7.4. Epirubicin (Farmorubicin RD®), EPI, was purchased from Pfizer (Ciudad de México, México) and was dissolved in sterile water for injection. Cyclophosphamide (Cryofaxol®), CTX, was purchased from Cryopharma (Tlajomulco de Zuñiga, Jalisco, México) and was dissolved in complete DMEM-F12 or RPMI. All stock solutions were wrapped in aluminum foil and stored at −20 °C.

### Cell culture

Murine microglia SIM-A9 (ATCC® HTB-22™) and murine breast cancer 4T1 (ATCC® HTB-26™) cells were purchased from the American Type Culture Collection (ATCC) and cultured in complete DMEM-F12 or RPMI for 4T1 and were routinely grown in 25-cm^3^ cell culture flasks (CORNING Enterprises, Corning, NY), following ATCC instructions. Cells were maintained in a humidified incubator containing 5 % CO_2_ at 37 °C. 

### Cell viability assessment

Cell growth inhibition was determined with Alamar blue (resazurin test). Cells (5x10^3^) were seeded in flat bottomed-96-well microtiter plates and exposed to different concentrations of EPI and CTX for 24 h. According to manufacturer's instructions, 20 μL of resazurin solution (0.15 mg/mL, Sigma-Aldrich, St. Louis, MO) was added to each well and incubated for 4 hours at 37 °C, after which, fluorescence was measured at 590 nm using a microplate reader (Varioskan Lux, Thermofisher®).

### Cell death analysis

For cell death induction, 5x10^4 ^cells were seeded in 24-well plates (CORNING). Cells were treated with the indicated concentrations of EPI or CTX and incubated for 24 hours at 37 °C. SIM-A9 cells were then detached using glucose (1 mg/mL), EDTA (1 mM) and EGTA (1 mM) solution trypsin (GIBCO) was used for 4T1 cells. Subsequently, cells were washed with PBS and resuspended in 100 μL of annexin binding buffer (100 mM HEPES pH 7.4, 1.4 M NaCl, and 25 mM CaCl_2_) staining cells with annexin-V-APC (0.1 μg/mL, BD Biosciences). Cells were then assessed with BD Accury 6 flow cytometer and analyzed using FlowJo Software.

### Cell cycle analysis

In brief, 2x10^5^ cells in 6-well dishes (CORNING) were incubated with the indicated concentrations of EPI or CTX for 24 h. Cells were then washed and fixed in 70 % ethanol (30 % PBS). Cells were washed again, and cell cycle distribution was determined by DNA staining. Cells were treated simultaneously with 25 μg/mL propidium iodide (PI) and 50 μg/mL RNase at 37 °C for 20 min. For DNA degradation, we analyzed the SubG1 population obtained from cell cycle analysis using flow cytometry. Cell DNA contents were then analyzed using FlowJo Software.

### Cell death-morphology analysis

After 5x10^4^ cells were seeded in 24-well dishes (CORNING) and treated with both chemotherapies at CC_50_ for 24 hours, cells were then observed in an inverted microscope (Nikon Eclipse TS100) and images were captured for assessing changes in morphology (Lummera INFINITY 1-2 CMOS 2.0 MP Camera) with 20x objective in bright-field.

### γ-H2AX, p53 and p16 assessment

Cells were seeded (2x10^5^) in 6-well dishes (CORNING) and incubated after treatment with cytotoxic concentration 50 (CC_50_) for 24 hours. After incubation, cells were detached, washed with PBS, and fixed with 80 % methanol (20 % PBS) and stored overnight at -20 ºC. Subsequently, cells were washed with 2 % FACS buffer and then rehydrated with 10 % FACS buffer and cells were placed at 4 ºC for 30 minutes. 

For γ-H2AX, p53 and p16 assessment, ab26350 mouse monoclonal [9F3] to gamma H2A.X (phospho S139. Abcam, Cambridge, UK) antibody, sc-126 DO-1 antibody (Santa Cruz, TX, USA) or p16 INK4a (ab108349), respectively, were incubated in 2 % FACS buffer in constant agitation for 1 hour and washed with 2 % FACS buffer. Therefore, a secondary antibody Alexa Fluor 488 (ab150077) for γ-H2AX and p16 or Alexa Fluor 488 (A11001. Thermo Fisher Scientific, Waltham, MA, USA) for p53 were added in constant agitation for 30 minutes and washed with 2 % FACS buffer. Cells were then assessed with BD Accury 6 flow cytometer and analyzed as mentioned before.

### Loss of mitochondrial membrane potential analysis

For this assessment, 5x10^4^ cells were seeded in 24-well dishes (CORNING) and then treated with CC_50_ of CTX or EPI during 24 h. Cells were then harvested and washed with PBS. DIOC_6_ (0.1 μM) (Invitrogene) staining was used by incubating cells at 37 °C for 30 min and washing them twice with PBS. Cells were then assessed by flow cytometry and analyzed using FlowJo Software.

### ROS production analysis

Cells were seeded (5x10^4^) in 24-well dishes (CORNING) and incubated with each chemotherapy at CC_50_ for 24 hours. Cells were then collected and washed with PBS. ROS generation was measured using DCFDA-AM (20 mM) (Thermo Fisher Scientific) staining that was incubated at 37 °C for 30 min. To evaluate the role of ROS in cell death, cells were pre-treated with N-acetyl-cysteine (NAC) for 30 minutes, then, cells were treated with CC_50_ of EPI or CTX. After 24 hours, cells were detached and resuspended in 100 μL of annexin binding buffer (100 mM HEPES pH 7.4, 1.4 M NaCl, and 25 mM CaCl_2_) staining cells with annexin-V-APC, after which, cell death was evaluated by flow cytometry.

### Autophagy assessment

SIM-A9 and 4T1 cells were seeded in 24-well dishes (CORNING) at a confluence of 5x10^4 ^cells/well, and incubated with CC_50_ of EPI or CTX alone or pre-treated for 30 min with spautin-1 (Abcam) (autophagy inhibitor). Cells were then harvested and stained using CYTO ID (ENZO technologies) following the manufacturer's instructions. Cells were then evaluated by flow cytometry and analyzed using FlowJo Software.

### Caspase activation assessment

SIM-A9 and 4T1 cultures were prepared by seeding 5x10^4 ^cells/well in 24-well dishes (CORNING), then incubated with EPI or CTX alone or pre-treated for 30 min with 10 mM QVD-Oph (Abcam) (pan caspase inhibitor). Cells were then detached and stained using a General Caspase Activation Kit (TF2-VAD-FMK) (Abcam) following the manufacturer's instructions. Caspase activity was determined by flow cytometry and analyzed using FlowJo Software.

### NO production analysis

For NO assessment, cells were treated with sub-lethal concentrations of EPI, after 24 h cells were detached and incubated at 37 ºC for 30 minutes with DAF-FM (ThermoFisher Scientific) and washed twice with PBS. Increases in cell fluorescence were measured by flow cytometry and analyzed using FlowJo Software.

### Cytokine quantification 

Cytokines in supernatants were analyzed with the CBA Mouse Inflammation kit (Beckman Dickinson and Company, Franklin Lakes, NJ) according to the manufacturer instructions. SIM-A9 cells, were seeded at a confluence of 5x10^4^ cells/well in 24-well dishes (CORNING) subsequently incubated for 24 h with sub-lethal concentrations of EPI. Supernatants were then collected and analyzed by flow cytometry using BD Accury 6 flow cytometer and were quantified using Cytometric Bead Array Analysis Software (FCAP) (Beckman Dickinson and Company).

### Iba-1 expression assessment

Cells were seeded (2x10^5^) in 6-well dishes (CORNING) and treated with both chemotherapies at CC_50_ for 24 hours. After incubation, cells were detached, washed with PBS and fixed with 80 % methanol (20 % PBS) and stored overnight at -20 ºC. Subsequently, cells were washed with 2 % FACS buffer and then rehydrated with 10 % FACS buffer and cells were placed at 4 ºC for 30 minutes. For Iba-1 expression polyclonal antibody (PA5-27436 Invitrogene, Thermo Fisher Scientific, Waltham, MA, USA) was incubated in 2 % FACS buffer in constant agitation for 1 hour and washed with 2 % FACS buffer. Then, a secondary antibody Alexa Fluor 488 (ab150077) was added in constant agitation for 30 minutes and washed with 2 % FACS buffer. Cells were then assessed with BD Accury 6 flow cytometer and analyzed as mentioned before.

### Statistical analysis

Results represent the mean ± SD of at least triplicate determinations from three independent experiments. Significant differences were considered if p<0.05 using paired student's t-test. Data was analyzed using GraphPad Prism (San Diego, CA). 

## Results

### Microglia cells are more sensitive to chemotherapy than breast cancer cells

**Figure 1:**
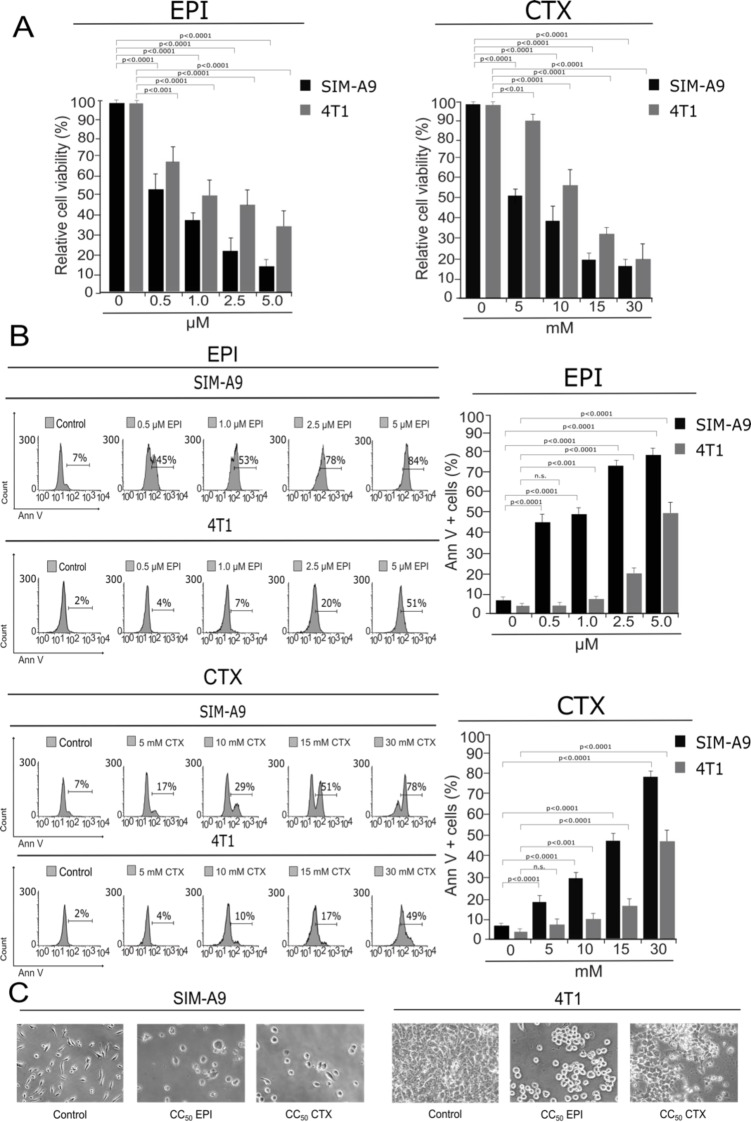
Cytotoxicity induced by EPI and CTX in SIM-A9 and 4T1 cells. SIM-A9 and 4T1 cells were treated with various concentrations (0.5, 1.0, 2.5 and 5.0 µM) of EPI, and 5, 10, 15 and 30 mM of CTX for 24 hours. A) Cell viability was measured by resazurin assay represented as percentage of control (non-treated cell viability = 100 %) presenting means ± SD. B) Cell death was measured by flow cytometry through Annexin-V staining. The histograms refer to Annexin-V positive cells analyzed by FlowJo software (left) and the bar graphs represent the mean (± SD) (right). C) Morphology assessment was performed with Nikon Eclipse TS100 using 20x objective.

EPI and CTX have been shown to suppress cell viability in several tumor cell lines (Trebunova et al., 2012[41]; Xiong et al., 2016[48]). However, their effect on cell viability and cell death on microglia cells has not been assessed, thus, we determined the effect of CTX and EPI in murine SIM-A9 microglia cells using 4T1, murine BC cells, as a control. Our results show that EPI and CTX decreased the viability of SIM-A9 and 4T1 cells in a dose-dependent manner (Figure 1A(Fig. 1)), however a higher concentration of chemotherapy was necessary to induce the same cytotoxic effect on BC cells. 

We further evaluated cell death by assessing phosphatidylserine (PS) exposure (Figure 1B(Fig. 1)). In healthy cells, PS is generally restricted to the inner leaflet of the cell membrane, and the exposure of phosphatidylserine on the outer leaflet is an effect that is commonly observed during cell death (Reyes-Ruiz et al., 2015[34]). We determined PS externalization by flow cytometry of Annexin-V-APC-labelled cells that were treated with EPI and CTX at different doses for 24 h. As shown in Figure 1B(Fig. 1), EPI and CTX induced cell death on SIM-A9 at significantly lower doses compared to 4T1 cells according to population of Annexin-V positive. Furthermore, as expected with resazurin results, EPI and CTX induced cell-death in a concentration-dependent manner. EPI induced cell death in 50 % of the cells (CC_50_) at 1.0 µM in microglia cells, and 5 µM in breast cancer cells, while CTX induced 50 % of cell death (CC_50_) at 15 mM in SIM-A9 and 30 mM in 4T1 cells. The CC_50 _for both chemotherapies were confirmed by trypan-blue staining (see Supplementary information). Moreover, morphological assessment showed a reduction of cell confluence and alterations in cell morphology that were visible after 24 hours of EPI and CTX treatment (Figure 1C(Fig. 1)).

### EPI and CTX induce DNA damage and cell cycle arrest

**Figure 2:**
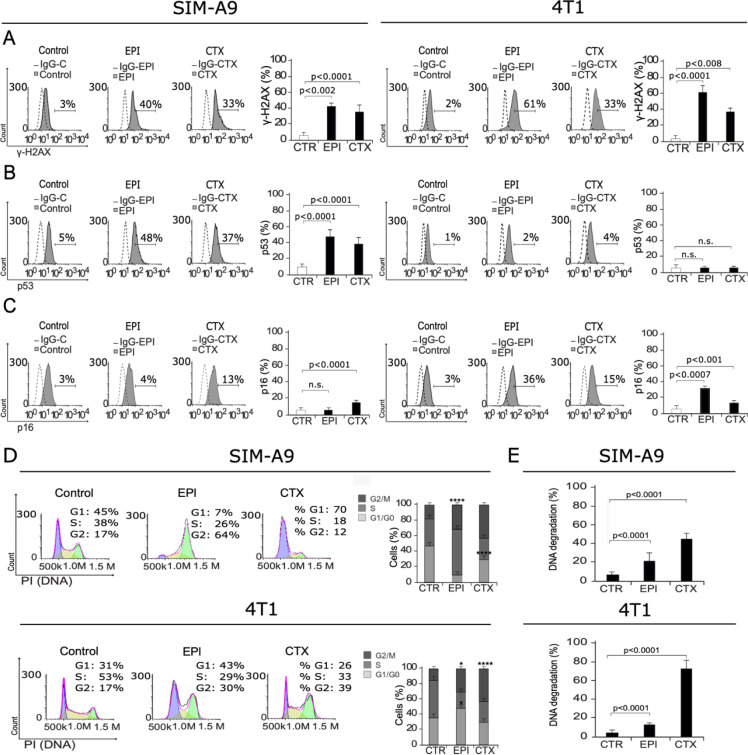
DNA damage and cell cycle arrest. SIM-A9 and 4T1 cells were treated for 24 h with CC_50_ of EPI or CTX and assessed by flow cytometry. Representative histograms of nuclear damage analysis and quantification measured through A) γ-H2AX assessment, B) p53 expression, C) p16 expression, and D) Cell cycle analysis. Bars at right represent the mean ± SD. E) DNA degradation was evaluated using propidium iodide (PI) staining as in D, and SubG1 population was analyzed and mean (± SD) was presented in bar graphs.

Once we observed cell death induction in SIM-A9 and 4T1 cells after EPI and CTX treatment, we decided to elucidate the mechanism. First, to evaluate if DNA damage was taking place after chemotherapy treatment of microglia cells, we first assessed H2AX phosphorylation (γ-H2AX). DNA damage can lead to γ-H2AX, the first step in recruiting and localizing DNA repair proteins (Mah et al., 2010[21]), which can initiate cell cycle arrest, followed by cell death and DNA degradation. We found that treatment with EPI and CTX increase the percentage of γ-H2AX-positive cells from 3 % to 40 % and 33 % respectively in SIM-A9 (left) and from 2 % to 61 % and 33 % respectively in 4T1 (right) cells (Figure 2A(Fig. 2)), indicating that both treatments induce DNA damage. Furthermore, we assessed p53 expression, and results show an increase on p53 expression for both treatments in SIM-A9, from 5 % to 48 % and 37 % for EPI and CTX, respectively (Figure 2B(Fig. 2)), by the time that p16 assessment showed a significant increase in CTX treatment in SIM-A9 cells (from 3 % to 13 %), while in 4T1, EPI and CTX treatment respectively, from 3 % to 36 % and 15 % (Figure 2C(Fig. 2)).

We then assessed cell cycle in microglia and breast cancer cells using PI staining. Our analysis shows that EPI induces cell cycle arrest on G2 in SIM-A9 and G1 and G2 in 4T1 cells, by the time that CTX induces cell cycle arrest on G1 in SIM-A9 and G2 in 4T1 cells, compared to control cells (Figure 2D(Fig. 2)).

As DNA degradation is a recurrent feature of cell death, especially after DNA damage (Kawane et al., 2014[18]), we analyzed DNA degradation by quantification of sub-G1 population in microglia and breast cancer cells treated with EPI and CTX. Results exhibit 22 % and 45 % of DNA degradation in SIM-A9 corresponding to EPI and CTX-24 hours treatment, respectively. In 4T1 cells DNA degradation relation was 17 % for EPI and 65 % in CTX for 24 hours treatment (Figure 2E(Fig. 2)). DNA degradation has been reported as a feature of apoptosis, hence, we decided to inquire in other apoptotic features such as loss of mitochondrial membrane potential and caspase activation.

### EPI and CTX induce loss of mitochondrial membrane potential and ROS production

**Figure 3:**
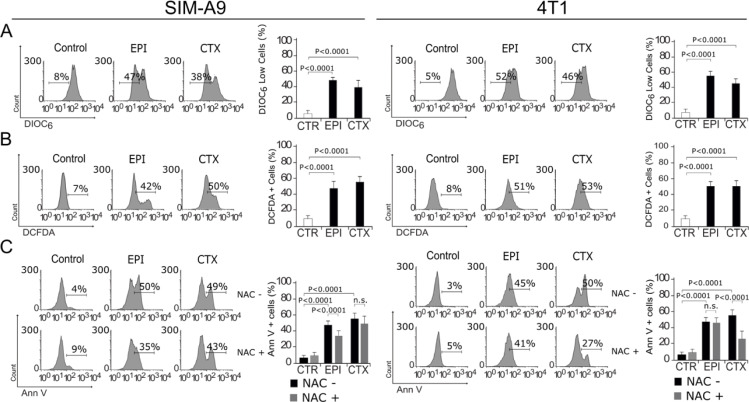
Mitochondrial alterations and ROS production. SIM-A9 and 4T1 cells were left without treatment (control) or treated with CC_50_ of EPI and CTX for 24 h and assessed by flow cytometry. A) Representative histogram of mitochondrial membrane potential measured by DIOC6 staining (left), bars represent the mean ± SD (right). B) Representative histogram of ROS production measured with DCFDA (left), bars represent the mean ± SD (right). C) Representative histogram of Annexin V staining, in cells pre-treated or not with the antioxidant NAC and then treated with EPI or CTX (left), bars represent the mean ± SD (right).

The role of mitochondria in cell death is widely known, as they play a central role in cellular metabolism and cell death signaling. Furthermore, mitochondrial dysfunction leads to reactive-oxygen species (ROS) production (Vakifahmetoglu-Norberg et al., 2017[43]). We assessed whether EPI and CTX were able to induce loss of mitochondrial membrane potential (ΔΨm) and ROS production, through 3,3'-Dihexyloxacarbocyanine Iodide (DIOC6) and 2′,7′-dichlorofluorescin diacetate (DCFDA) staining, respectively, followed by flow cytometric analysis. As shown in Figure 3A(Fig. 3), EPI and CTX induce ΔΨm loss and ROS production, as shown by flow cytometry (Figure 3B(Fig. 3)) in both cell lines. We also found a correlation between ROS production and PS exposure, because approximately 50 % of the cells display these two features (Figure 3C(Fig. 3)). Then, we used the antioxidant N-acetyl-L-cysteine (NAC), which increases intracellular glutathione (GSH) levels and possesses thiol-disulfide exchange activity (Xie et al., 2018[47]), to determine if ROS were playing a role in chemotherapies-induced cell death, pre-treating cells with NAC. As shown in Figure 3C(Fig. 3), NAC was able to inhibit EPI induced cell death in SIM-A9 cells, as observed by the reduction of Annexin V+ staining. These results show that EPI and CTX induce ΔΨm loss and ROS production, and particularly after EPI treatment we observed ROS-dependent cell death in SIM-A9. On the other hand, we observed ROS production in 4T1 cells treated with CC_50_ of EPI and CTX. Using NAC, we determined ROS dependency for cell death in 4T1 cells after CTX treatment, but not after EPI's treatment.

### EPI and CTX induce autophagy

**Figure 4:**
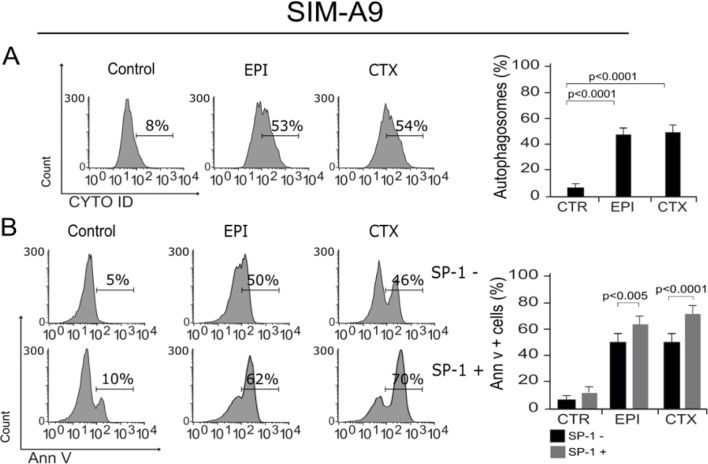
Autophagosome formation and cell death. A) Representative histogram of autophagy, measured by flow cytometry using Cyto-ID staining in SIM-A9 cells treated with EPI and CTX for 24 hours (left), bars represent the mean ± SD (right). B) Representative histograms showing autophagy involvement in cell death, assessed by pre-treating SIM-A9 cells with the autophagy inhibitor, SP-1, and measuring cell death by Ann V-positive cells after 24 hours of treatment with EPI or CTX (CC_50_) (left), bars represent the mean ± SD (right).

It has been proven that ROS and ΔΨm loss could lead to autophagy, a protective mechanism that helps cells survive and has been previously described as a regulatory mechanism in microglia cells (Plaza-Zabala et al., 2017[30]). In this regard, we assessed autophagosome formation in microglia cells after treatment with EPI and CTX. As shown in Figure 4A(Fig. 4), both chemotherapies induce autophagy at CC_50_. Furthermore, we evaluated cell death in cells pre-treated with spautin-1 (SP-1) an autophagy inhibitor by enhancing degradation of beclin-1 (Schott et al., 2018[35]), to confirm if the autophagy was playing a protective role. We observed that SP-1 pre-treatment increased the cell death induced by the CC_50_ of both chemotherapies (Figure 4B(Fig. 4)). 

### EPI and CTX induce caspase activation

**Figure 5:**
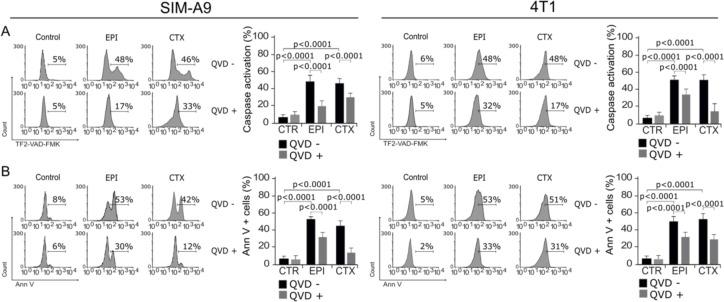
Activation and role of caspases in cell death. A) Representative histograms of caspase activation measured by flow cytometry using TF2-VAD-FMK in SIM-A9 and 4T1 cells treated with EPI and CTX for 24 hours (left). Bars at right represent the mean ± SD. B) Cells were pretreated with the pan-caspase inhibitor QVD-Oph and then treated with EPI or CTX (CC_50_) for 24 hours and Ann V positive SIM-A9 or 4T1 cells were assessed. Representative histograms (left) and bar graphs presenting mean ± SD (right) are shown.

To evaluate if the main molecular regulators of apoptosis were activated by EPI and CTX, we assessed caspase activity (McIlwain et al., 2015[24]) after treatment. As shown in Figure 5(Fig. 5), EPI and CTX induce caspase activation, as determined by the detection of TF2-VAD-FMK (Figure 5A(Fig. 5)). To determine if this type of cell death was dependent on caspase activity, we used the pan-caspase inhibitor, QVD-Oph (Keonie and Brown, 2015[19]). As Figure 5A(Fig. 5) shows, QVD was able to inhibit caspase activation in SIM-A9 and 4T1 cells. Furthermore, we found that chemotherapies-induced cell death was diminished in presence of QVD (Figure 5B(Fig. 5)). This result shows that EPI and CTX induce caspase-dependent cell death in both cell lines.

### EPI's low concentration induces DNA damage and cell cycle arrest

Studies show that EPI and CTX have limited to none ability to cross blood brain barrier (BBB) (Guo et al., 2011[17]), a selective protective membrane that covers blood vessels that cross the central nervous system (Vieira and Gamarra, 2016[45]), however, anthracyclines and alkylating agents are able to disrupt BBB after a certain number of chemotherapy cycles (Ren et al., 2019[32]). Considering the quantity of chemotherapy needed to achieve CC_50_, we decided to test low concentrations of EPI and CTX to determine whether these concentrations induce DNA damage and further cell cycle arrest. For this, we used sub-lethal (SL) concentrations and CC_20_ of EPI (SL of 0.1 µM and CC_20_ of 0.25 µM), and CTX (SL of 2 mM and CC_20_ of 5 mM) in microglia cells. 

**Figure 6:**
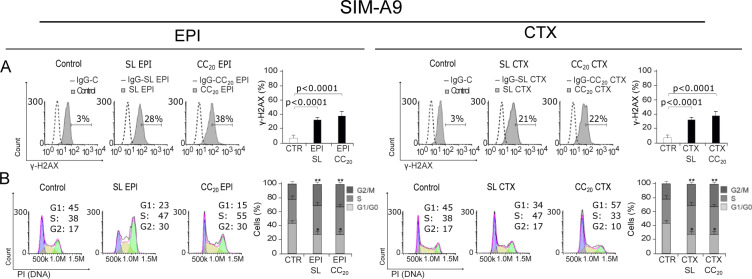
DNA damage and cell cycle arrest induced by low concentrations of EPI and CTX. A) Representative histograms of nuclear damage analysis and quantification measured through γ-H2AX. Bars at right represent the mean ± SD. B) Cell cycle analysis by propidium iodide (PI) staining after treatment with SL or CC_20_ of EPI or CTX in SIM-A9 for 24 h. Representative histograms (left) and bar graphs presenting mean ± SD (right) are shown.

Our results show that EPI can induce DNA damage (Figure 6A(Fig. 6)) and cell cycle arrest (Figure 6B(Fig. 6)) at SL and CC_20_. On the other hand, CTX did induce DNA damage at SL concentration (Figure 6A(Fig. 6)), but no cell cycle arrest was observed, although G1 arrest was observed at CC_20_ (Figure 6B(Fig. 6)).

### EPI induces microglia activation

**Figure 7:**
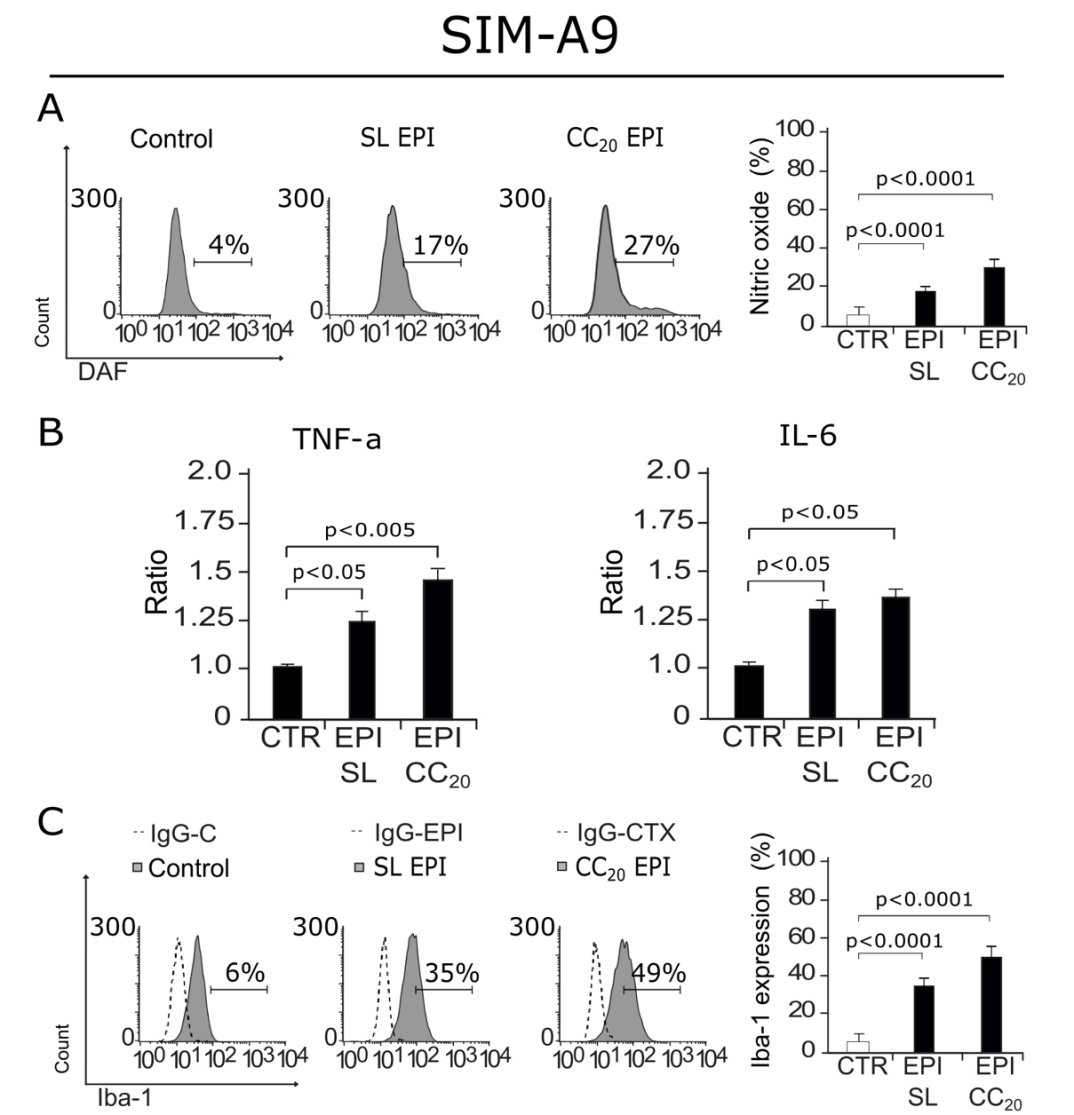
NO production, cytokine release and Iba-1 expression after SIM-A9 treatment with EPI. A) Representative histograms of Nitric oxide (NO) (left) production measured through DAF staining, bar graphs presenting mean ± SD (right) are shown. B) Bar graphs displaying mean ± SD of TNF-α and IL-6 cytokine release assessed in SIM-A9 cells treated for 24 hours with sub-lethal (SL) concentration of EPI or EPI CC_20_. C) Representative histograms of Iba-1(left) expression assessed by anti-Iba-1 antibody, bar graphs presenting mean ± SD (right) are shown.

As our results show DNA damage and cell cycle arrest from a sub-lethal concentration, we decided to evaluate if EPI was also able to induce the production of the neuroinflammatory mediator, nitric oxide (NO) (Frank et al., 2019[10]). In Figure 7A(Fig. 7), we can observe an increase in NO production in cells treated with EPI at a SL concentration and EPI CC_20_. Then, as cytokines are key players in neuroinflammation, and as an increase in p16 and γ-H2AX have been related to cytokine production by microglia (Marques et al., 2020[22]) we next assessed cytokine release after EPI (SL and CC_20_) treatment. Our results show that EPI induces a significant increase in TNF-α and IL-6 release at SL and CC_20_ (Figure 7B(Fig. 7)). These results led to the idea that microglia cells could have been activated by EPI treatment, to solve that we assessed Iba-1 (ionized calcium-binding adapter molecule 1) expression, as its overexpression is correlated to microglia activation (Zhao et al., 2019[52]). In Figure 7C(Fig. 7) we observe that EPI increases Iba-1 expression in cells treated with SL and CC_20_ concentrations, indicating that EPI's treatment induces microglia activation.

## Discussion

The objective of this study was to analyze the effects induced by EPI and CTX in microglia cells, comparing sensitivity and cell death mechanisms between both cell types. The results shown in this study were noteworthy, starting with the fact that SIM-A9 cells are highly susceptible to EPI and CTX, approximately five times more for EPI and two times more for CTX than 4T1. There have been several studies showing the response of microglia with chemotherapies, but none for them had addressed the type of cell death induced by EPI and CTX. Here we show that both chemotherapies induce DNA damage, as shown by γH2AX, an histone variant that respond to double strand breaks (Kitazumi and Tsukahara, 2011[20]), that has also been described as a senescent marker (Noren Hooten and Evans, 2017[28]). To assess activation of repair genes, p53 and p16 were evaluated, since 4T1 cells are p53 null (Yerlikaya et al., 2012[50]), hence p16 assessment was important to compare proteins capable of inducing cell cycle arrest. Increase in p53 activation and in p16 was observed in SIM-A9 cells, according to cell cycle arrest in G1 and in G2 phase by EPI and CTX-treatment, respectively. Some chemotherapies are able to activate and produce p53 and p16 (Marques et al., 2020[22]) in microglia, however, p16 and γH2AX are also senescent markers, pointing out that more research must be made to determine if EPI and CTX induce senescence in microglia cells, which is known that can lead to neurodegeneration (Angelova and Brown, 2019[2]).

It is well known that most chemotherapies are able to disrupt mitochondrial membrane potential (Gorini et al., 2018[15]), our findings suggest that ΔΨm loss in microglia occurs and could lead to oxidative stress, which is known to occur in microglia and in tissue of *in vivo* models treated with doxorubicin (DOX) (Cruzado et al., 2014[5]), methotrexate (MTX) (Gibson et al., 2019[14]), and some other chemotherapies (Gaman et al., 2016[12]), but the role of ROS in cell death has not been described before. We observed a dependence of ROS for cell death in SIM-A9 treated with EPI. Anthracyclines increase ROS production to induce cell death but, oxidative stress could lead to a direct disruption in CNS homeostasis, generating neurotoxicity and diminishing cognitive performance in patients (Bergamini et al., 2018[3]; Solleiro-Villavicencio and Rivas-Arancibia 2018[38]; Misra et al., 2020[26]). As we demonstrated autophagy induction after chemotherapy treatment (Figure 4(Fig. 4)), cytotoxicity of both chemotherapeutic agents could be reduced by pro-survival autophagy inductors as it has been described before in *in vivo* studies (Yi et al., 2020[51]). Also, our assessment of caspase activity showed evidence of caspase activation in both treatments over microglia and breast cancer cells, as well as their dependency for cell death. These results lead to the conclusion that apoptotic cell death was happening in microglia after 24 hours of EPI CC_50_ and CTX CC_50_ treatment.

Although it is widely known that CTX is not able to cross BBB, there is no information for EPI, however, DOX has limited capacity to penetrate BBB (Vieira and Gamarra, 2016[45]). Although scarce, there is evidence supporting chemotherapy increase BBB permeability, allowing the direct interaction between chemotherapies and microglia (Ren et al., 2017[33]). By analyzing how much chemotherapy is needed to achieve 50 % of cytotoxicity (CC_50_), we asked ourselves what would happen if lower concentrations of chemotherapy interact with microglia. Hence, we used sub-lethal (non-lethal) concentrations and CC_20_ of both agents. Results of γH2AX analysis show that both chemotherapies induce DNA damage at SL concentration and CC_20_. Furthermore, as some chemotherapies can disrupt the cell cycle at low concentrations, we evaluated if DNA damage could lead to cell cycle arrest, indeed, cells treated with EPI from SL concentrations to CC_50_ were found arrested in G2, whereas for CTX treated cells arrest G1 was observed at CC_20_ and CC_50_. These findings suggest that EPI's negative effects in microglia occur before cytotoxicity. Similar findings have been reported with DOX in microglia cells (Marques et al., 2020[22]).

Because EPI's negative effects in microglia were evident from SL concentrations, we decided to assess NO production at the lowest concentration of EPI where cell death was not observed. Increases in NO have been associated with neuroinflammation and augmenting oxidative stress in CNS (Frank et al., 2019[10]). As we observed, EPI at SL and CC_20_ can increase NO production. It has also been described that increases in TNF-α and IL-6 could be hurtful for the CNS and there is evidence of their induction by some chemotherapies (Ren et al., 2017[33]). As microglia cells are capable of releasing cytokines depending on their activation pathway, we assessed cytokine release in response to chemotherapy stimuli. We observed and increase in TNF-α and IL-6, at SL of EPI after 24 hours of treatment, this has been proven in microglia cells treated with DOX and could mean that these chemotherapies could have the capability to activate microglia and dysregulate cytokines in CNS that could potentially initiate neurodegeneration *in vivo* (Marques et al., 2020[22]). However, other microglial activation markers had to be assessed to conclude activation, hence, we assessed Iba-1 expression (ionized calcium-binding adapter molecule 1), as its overexpression is correlated with microglia activation (Zhao et al., 2019[52]). We observed that EPI's treatment induced Iba-1 overexpression. This result, along with NO production and pro-inflammatory cytokine release led us to conclude that EPI's treatment induces microglia activation, which has been associated with neurodegeneration and it's believed to be one of the mechanisms of CRCI (Monje and Dietrich, 2012[27]; Cerulla et al., 2019[4]; Gibson and Monje, 2019[13]; Du et al., 2021[7]).

It is also noteworthy that a very low concentration of EPI is needed to damage microglia or to induce their activation. Apoptosis induced by TNF-α released by activated microglia in progenitor neural cells (NPC) has been observed (Guadagno et al., 2013[16]), as well as cognitive decline in LPS stimulated-mice in *in vivo *models (Zhao et al., 2019[52]). Finally, IL-6 has not only been associated with neurodegeneration and senescence, but with loss of lean body and fat mass in tumor-free mice (Elsea et al., 2015[8]), suggesting the importance of assessing cytokine release by peripheral immune cells treated with chemotherapy and moreover, the interaction between the peripheral and central immune system. Thus, although further research must be done to assess these effects *in vivo*, here we demonstrate that EPI and CTX induce important cell effects in microglia, causing DNA damage since sub-lethal concentrations, while EPI induces microglia activation and a proinflammatory phenotype at low concentrations.

## Conclusions

**Figure 8:**
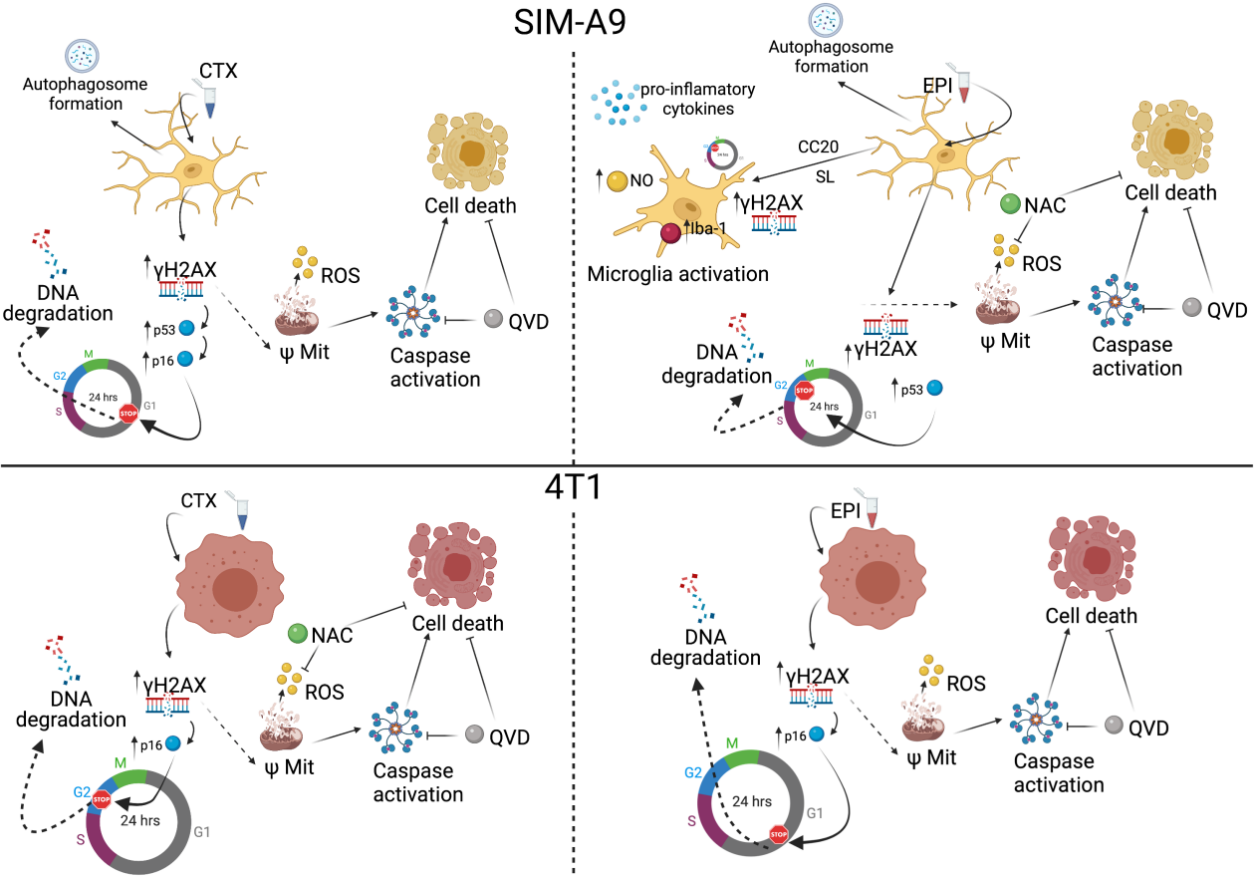
Chemotherapy effect in microglia and breast cancer cells. CTX induces DNA damage and cell cycle arrest, as well as mitochondrial alterations and increase in ROS production leading to ROS-dependent cell death in breast cancer cells (down left), but not in microglia cells (upper left). Caspase activation and caspase-dependent cell death are also observed in both cell lines. In microglia, CTX treatment induces autophagosome formation. EPI induces DNA damage and cell cycle arrest, as well as mitochondrial alterations and increase in ROS production leading to ROS-dependent cell death in microglia (upper right), but not in breast cancer cells (down right). Caspase activation and caspase-dependent cell death are also observed in both cell lines. Furthermore, EPI also induces autophagosome formation in microglia. Finally, CC_20_ and sub-lethal concentrations of EPI induce DNA damage, cell cycle arrest and microglia activation, increasing nitric oxide production and pro-inflammatory cytokine release in SIM-A9 cells.

In summary, the present research demonstrates that microglia cells are more susceptible to CTX and EPI than breast cancer cells (approximately two to five times with CTX and EPI respectively) and both chemotherapies induced DNA damage, cell cycle arrest, and apoptosis (Figure 8(Fig. 8)). Interestingly, low concentrations of EPI induced microglia activation, as demonstrated by NO production, pro-inflammatory cytokines release, IL-6 and TNF-α, and the overexpression of Iba-1 (Figure 8(Fig. 8)). Further research is needed to evaluate if this could happen *in vivo*. Also, this work opens the door to the study of new agents that can diminish microglia cell death and activation, as well as the identification of the mechanisms leading to these effects.

## Notes

Ana Carolina Martínez-Torres and Cristina Rodríguez-Padilla contributed equally as last author.

## Declaration

### Declaration of competing interest 

The authors have no conflicts of interest to declare.

### Acknowledgments 

This work was funded by PAICYT (UANL) (grant CN1563-21) and the Laboratorio de Inmunología y Virología (UANL).

## Supplementary Material

Supplementary information
