# Supplementary material for: Cyclophosphamide and epirubicin induce high apoptosis in microglia cells while epirubicin provokes DNA damage and microglial activation at sub-lethal concentrations
Source: EXCLI J. 2022 Jan 10;21:197–212. doi: 10.17179/excli2021-4160 (PMC8822306; doi:10.17179/excli2021-4160)
Supplement: Supplementary information [file EXCLI-21-197-s-001.pdf]

EXCLI Journal 2022;21:197-212 – ISSN 1611-2156
Received: September 22, 2021, accepted: December 13, 2021, published: January 10, 2022

Supplementary information to:

Original article:

CYCLOPHOSPHAMIDE AND EPIRUBICIN INDUCE HIGH APOPTOSIS IN MICROGLIA CELLS WHILE EPIRUBICIN PROVOKES DNA DAMAGE AND MICROGLIAL ACTIVATION AT SUB-LETHAL CONCENTRATIONS

Rafael de la Hoz-Camacho[a], Ana Luisa Rivera-Lazarín[a], Jose Manuel Vázquez-Guillen[a], Diana Caballero-Hernández[a], Edgar Mendoza-Gamboa[a], Ana Carolina Martínez-Torres[a,+*], Cristina Rodríguez-Padilla[a,b,+]

[a] Universidad Autónoma de Nuevo León, Facultad de Ciencias Biológicas, Laboratorio de Inmunología y Virología, Monterrey 66455, México
[b] LONGEVEDEN S.A. de C.V.

[+] These authors share equal co-seniorship.

* **Corresponding author:** Ana Carolina Martínez-Torres, Universidad Autónoma de Nuevo León, Facultad de Ciencias Biológicas, Laboratorio de Inmunología y Virología, Monterrey 66455, México. Tel +52 8 121 4115. Fax +52 818 352 4212.
E-mail: ana.martinezto@uanl.edu.mx

https://dx.doi.org/10.17179/excli2021-4160

This is an Open Access article distributed under the terms of the Creative Commons Attribution License (http://creativecommons.org/licenses/by/4.0/).

EXCLI Journal 2022;21:197-212 – ISSN 1611-2156
Received: September 22, 2021, accepted: December 13, 2021, published: January 10, 2022

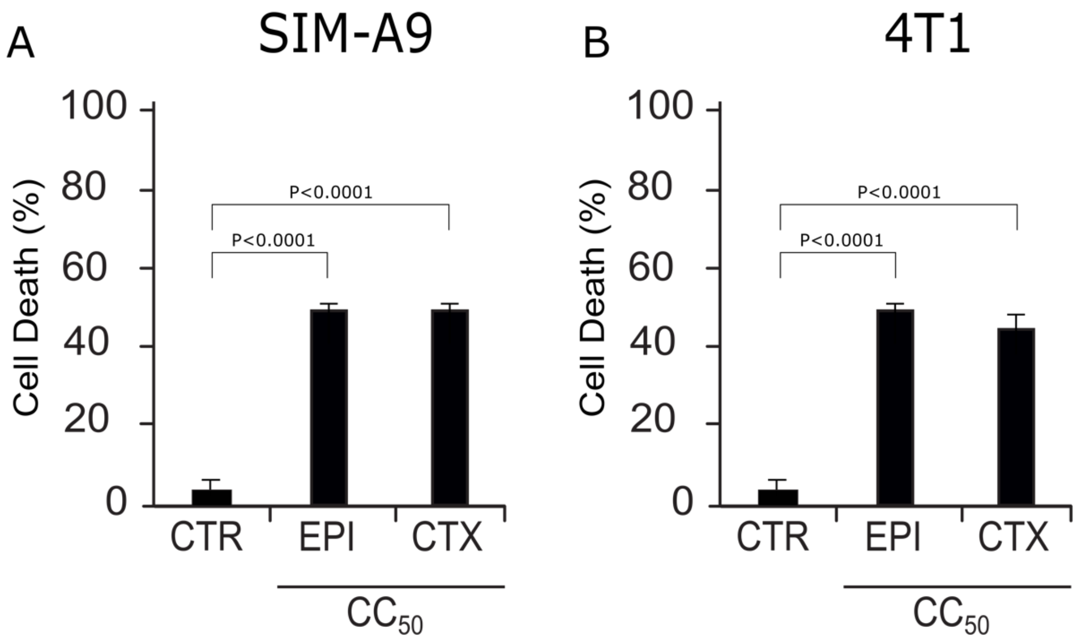

Supplementary Figure 1: Cell death induced by EPI and CTX in SIM-A9 and 4T1 cells assessed with trypan blue. Cell death was measured in SIM-A9 and 4T1 cells treated with EPI CC_{50} and CTX CC_{50} for 24 hours, after which, cells were counted with trypan blue. **A**) Bar graphs represent the mean (± SD) of cell death observed by trypan blue after treatment with EPI CC_{50} (1 µM) and CTX CC_{50} (15 mM) in SIM-A9 cells. **B**) Bar graphs represent the mean (± SD) of cell death observed by trypan blue after treatment with EPI CC_{50} (5 µM) and CTX CC_{50} (30 mM) in 4T1 cells.
